# Designing spiking neural models of neurophysiological recordings using gene expression programming

**DOI:** 10.1186/1471-2202-14-S1-P74

**Published:** 2013-07-08

**Authors:** Josafath I Espinosa-Ramos, Roberto A Vazquez, Nareli Cruz-Cortes

**Affiliations:** 1 Computation Research Center, National Polytechnic Institute, Mexico City, 0738, Mexico; 2 Intelligent Systems Group, Faculty of Engineering, La Salle University, Mexico City, 06140, Mexico

## 

Spiking Neural Models (SNMs) can accurately predict the spike trains produced by cortical neurons in response to somatically injected currents. Since the specific characteristics of the model depend on the neuron, a computational method is required to fit models to electrophysiological (EP) recording. However, important drawbacks of these models are that they only work within the defined limits to fit the EP recordings presented. These limitations suggest that the ideal would not be to fit existing models, but to construct a model for each kind of neurons. Recently, several labs around the world have approached the question about what constitutes a good neuron model by assessing it quality regarding to spike timing prediction or features of the voltage trace.

This work describes a first effort to design a methodology that creates automatically SNMs using an Evolutionary Computation Strategy (ECS). This methodology generates a mathematical equation that reproduces the behavior of biological neurons. Creating a SNM to reproduce EP data is performed by maximizing a fitness function which measures the adequacy of the model to the data. This task is done by using Gene Expression Programming (GEP), an ECS that automatically creates computer programs such as conventional mathematical models, sophisticated nonlinear models, and so on. In this research, we applied the gamma factor as a fitness function [1], which is based on the number of coincidences between the model spikes and the spikes experimentally recorded.

**Figure 1:**
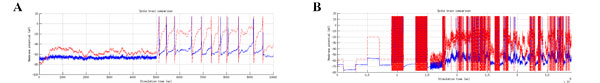
**Experimental results obtained with the generated equation (62.34/v^4^) + (v/(20.165- v))^2 ^+0.34**. In blue the reference signal, and in red dotted the signal generated with the previous equation. (a) Simulation within 1 s. (b) Simulation within 39 s.

In order to test the approach accuracy, we used the EP recordings launched by the International Neuroinformatics Coordinating Facility, specifically challenge B [2]. The training data consist of the injected currents and the pyramidal neuron voltage recordings where the digitization (time step) is 0.1 ms., that corresponds to a sampling frequency of 10 KHz. The current-clamp stimulus has two parts: the first part is 17.5 s of various waved stimulus, such as hyperpolarizing, depolarizing, and white noise; the second part of the stimulus takes the remaining 42.5 s, and is made of a simulated excitatory and inhibitory spike train [1]. In order to generate the SNM, we choose a sample of 1 s from the voltage sample that contains 0.5 s of white noise, and 0.5 s of simulated excitatory or inhibitory spike train. After applying GEP for 5000 generations, we obtained a mathematical model that describes the behavior of the pyramidal neuron shown in Figure 1.

Experimental results suggest that the proposed methodology can be applied to generate SNM from EP recordings with high accuracy. Although the signal shape is not the same compared with the reference signal, spike timing matched the reference signal with great accuracy.
